# Supplementary material for: Multidimensional characteristics of young Brazilian volleyball players: A Bayesian multilevel analysis
Source: PLoS One. 2021 Apr 30;16(4):e0250953. doi: 10.1371/journal.pone.0250953 (PMC8087100; doi:10.1371/journal.pone.0250953)
Supplement: S1 File — (PDF) [file pone.0250953.s001.pdf]

Supplementary figures for the manuscript "Multidimensional characteristics of young Brazilian volleyball players"

Felipe G. Mendes, Ahlan B. Lima, Marina Christofoletti, Carine Collet, Carlos E. Gonçalves and Humberto M. Carvalho

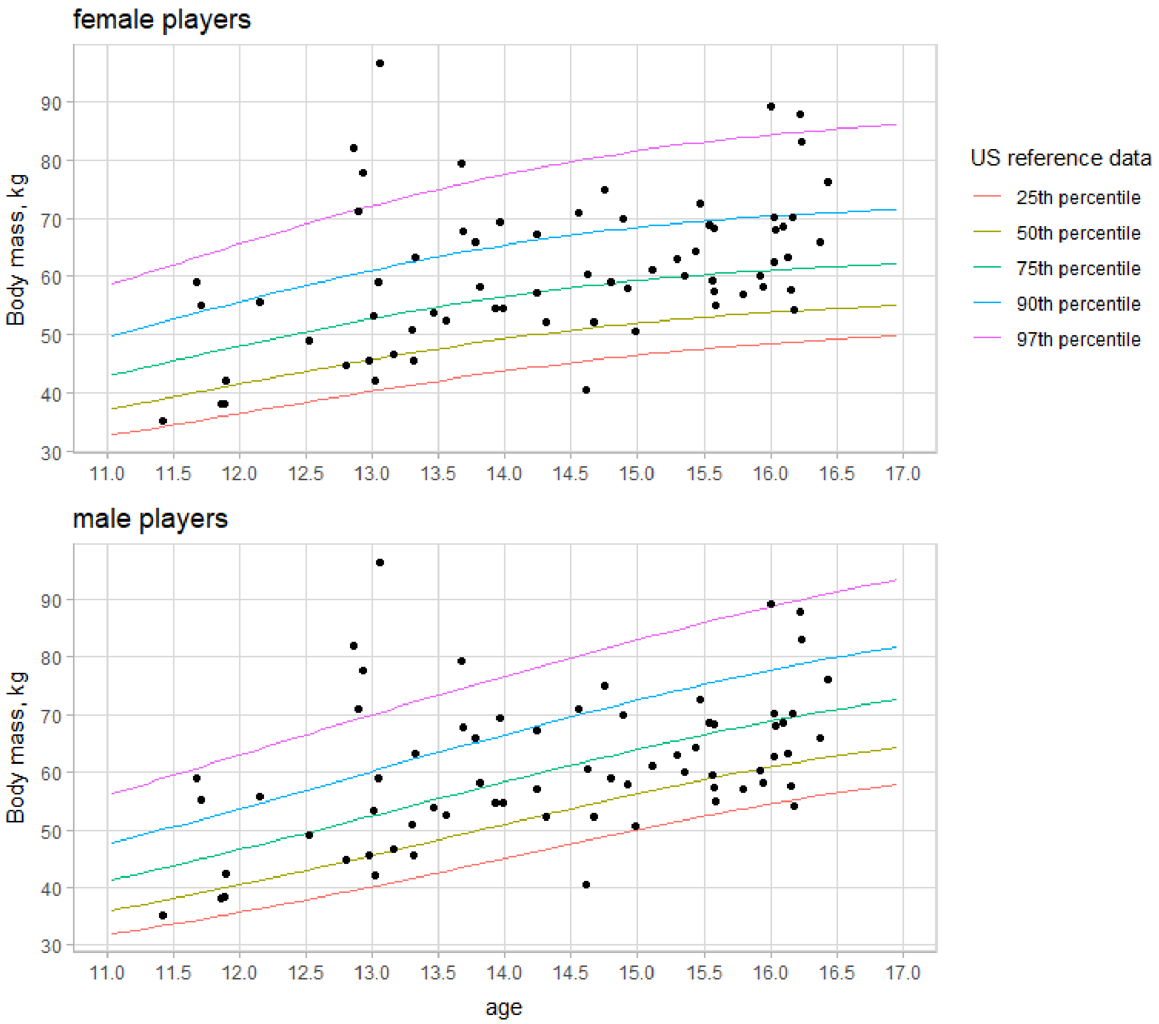

S1 fig. Masses of young female (upper panel) and male (lower panel) volleyball players by chronological age against the US population growth references for body mass.

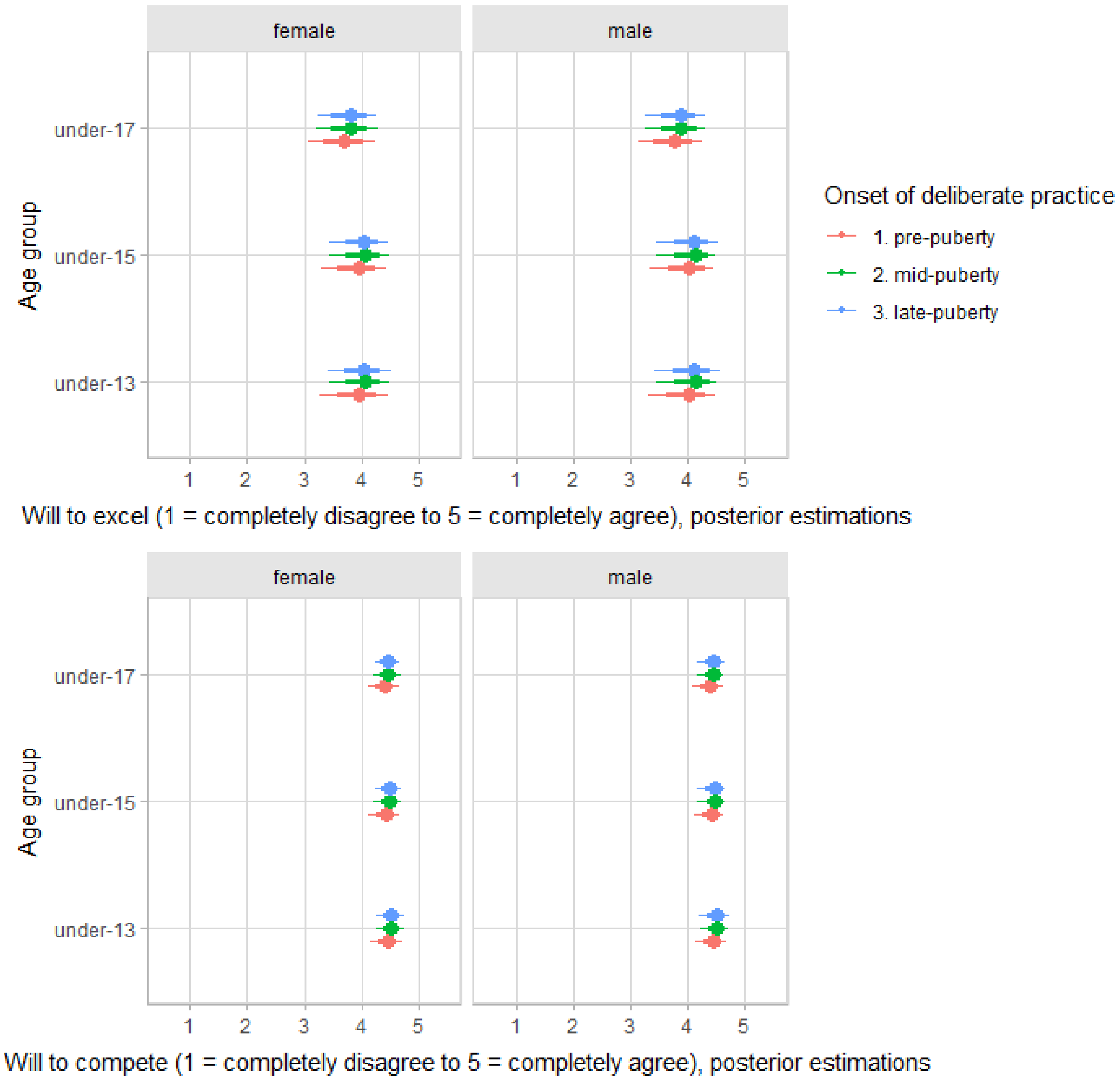

S2 fig. Posterior estimations and uncertainty (bold lines and thick ones represent 67% and 90% intervals, respectively) for will to excel (upper panel) and will to compete (lower panel) by age group and contrasting age group by the onset of deliberate practice.

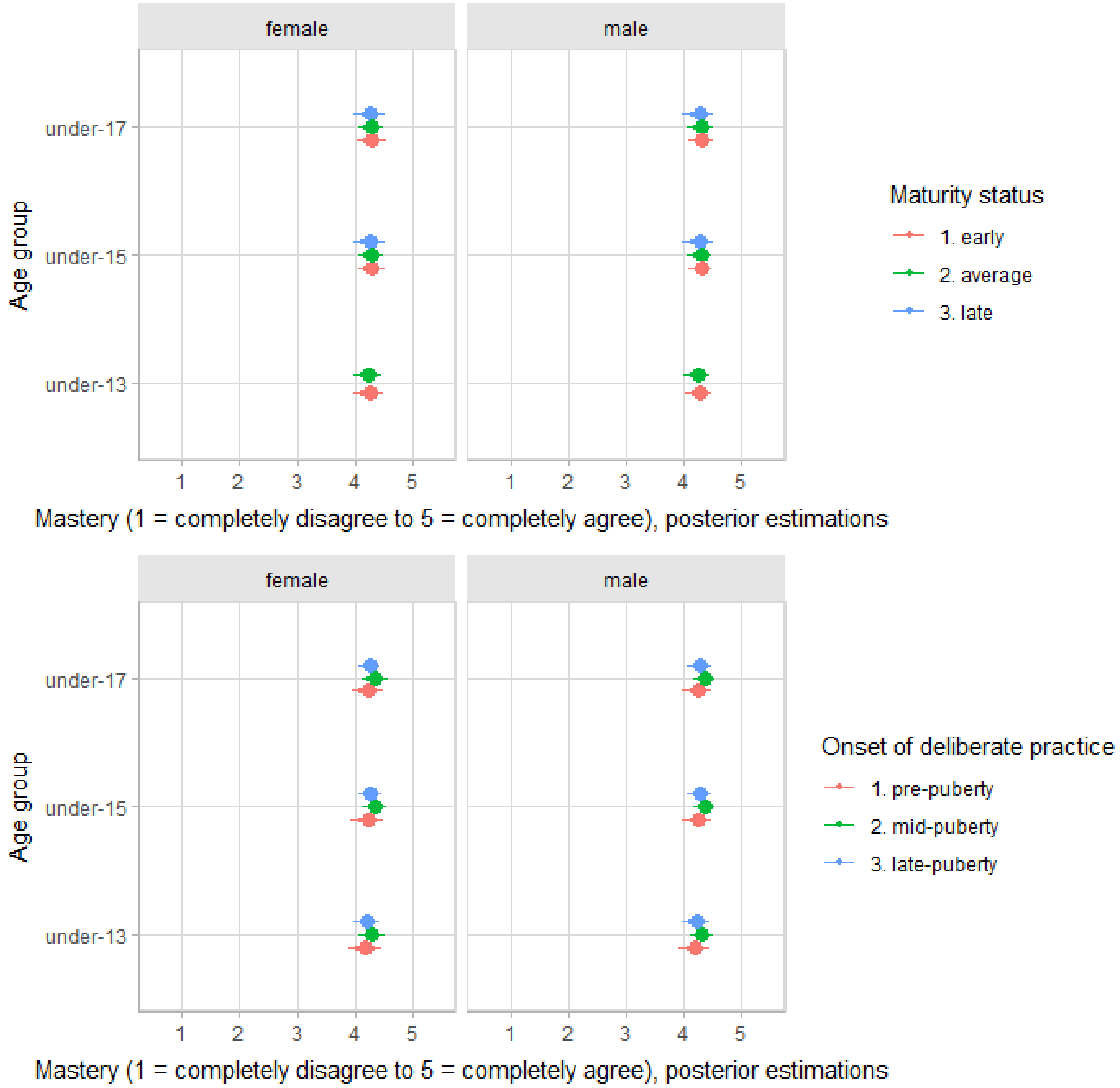

S3 fig. Posterior estimations and uncertainty (bold lines and thick ones represent 67% and 90% intervals, respectively) for mastery scores by age group and contrasting age group by maturity status (upper plots), and the onset of deliberate practice (lower plots).

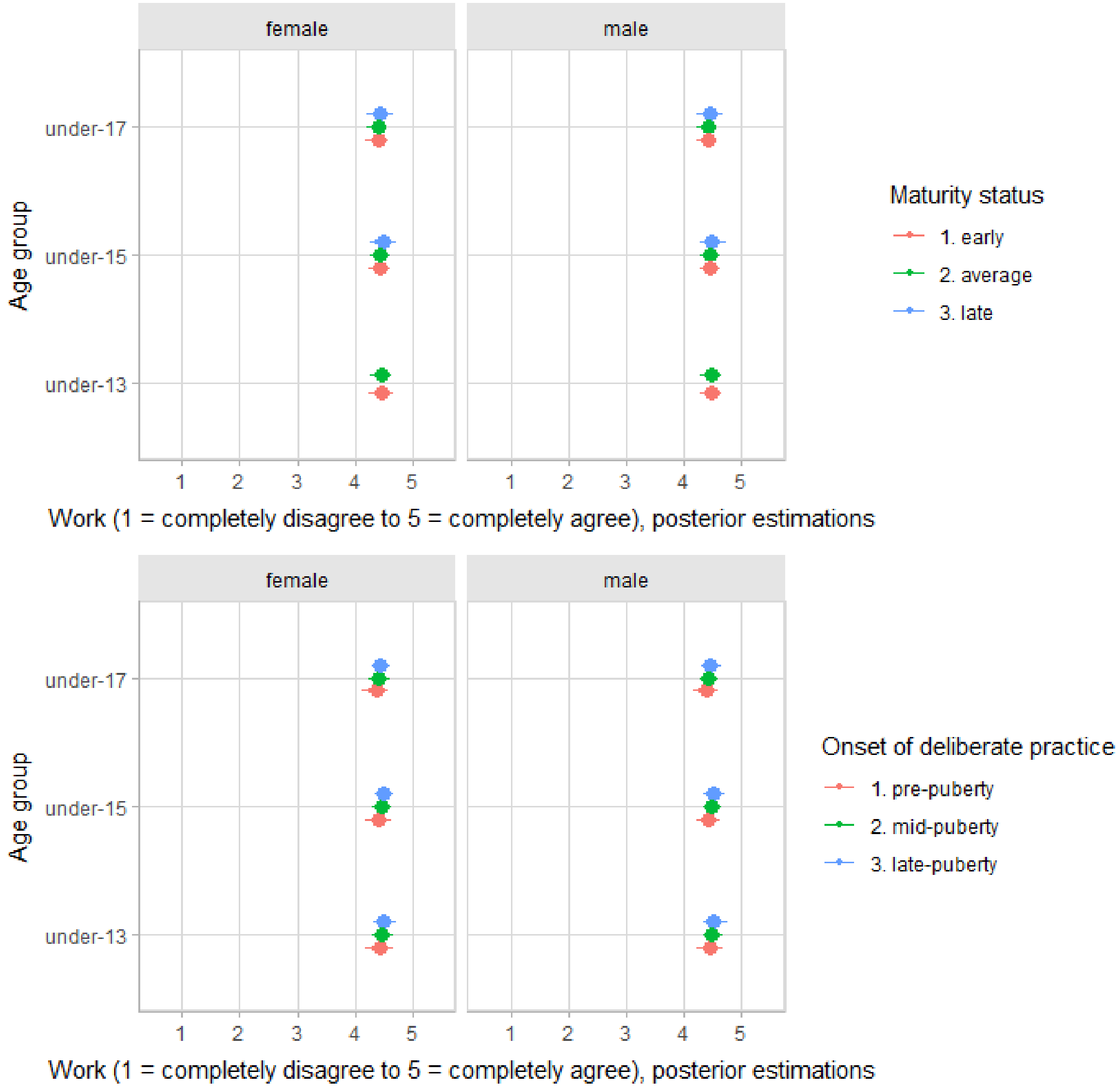

S4 fig. Posterior estimations and uncertainty (bold lines and thick ones represent 67% and 90% intervals, respectively) for work scores by age group and contrasting age group by maturity status (upper plots), and onset of deliberate practice (lower plots).
