# Supplementary material for: Multidimensional characteristics of young Brazilian volleyball players: A Bayesian multilevel analysis
Source: PLoS One. 2021 Apr 30;16(4):e0250953. doi: 10.1371/journal.pone.0250953 (PMC8087100; doi:10.1371/journal.pone.0250953)
Supplement: Attachment — Submitted filename: Response to Reviewers.docx [file pone.0250953.s002.docx]

**Specific reply to reviewers´ comments**

Authors´ reply: We appreciate both reviewers' comments, and hopefully, we satisfactorily addressed them.

Reviewer #1: l118: The abbreviated term, PHV, was observed at first time here, but you did not explain it beforehand. At line 135, I found that the “peak height velocity” was abbreviated to the PHV.
Authors´ reply: We corrected the first abbreviation of the term.

l123,-123, l138-l140, l146-147:
Same information of PHV is duplicated and redundant. Please consider if it should be omitted.

Authors´ reply: We agree with the reviewer. The information was redundant and removed.

l201:
How did you categorize age groups? In the equation, participants’ age seemed to be divided into four groups.However, I could not find the descriptions of divided points for age in the Method section.In figures, age groups were shown as under-17, under-15, and under-13. However, these were three age groups, not four.

Authors´ reply: We thank the reviewer for raising this point. The players were grouped into two-year age categories: under 13 (11.0-12.9), under 15 (13.0-14.9), and under 17 (15.0-16.9) years. Also, we corrected the equation as three groups should be represented.

l201, l202:
These symbols were mistakes?
for i = 1, 2, 3, 4 -> for a = 1, 2, 3, 4
for s = 1, 2,3 -> for d = 1, 2, 3
Authors´ reply: Again, thank you for noting the typos. We corrected the equation indexes.

l200-l214:
I could not understand well the reason why you applied a multilevel model for this data in which each group included only several categories.
For example, when you inferred the effect of gender on outcomes, it would be enough to estimate the fixed effect of the gender (i.e., slope of the gender on the outcome) using an ordinary regression model. At least, for binary variable (i.e., gender), it seemed to be unnecessary to assume the hierarchical structure of parameters.
In addition, you categorized age into some groups, but the effect of age on outcomes should be inferred using continuous predictive variable. If you pooled continuous age values into some categories, you would lack much information about influence of age on outcome.

Taken together, it seemed that the ordinary regression model would be enough for your analysis. If not so, please explain clearly the purpose of using multilevel model for this study.

Authors´ reply: We appreciate the reviewer's comment. Ordinary single-level regression would be innapropriate to deal with the data because:

(i) the context of observation that presents a cross-classified nesting, as players belong to multiple groups (eg, a late maturing boy from the under 13 age group with an onset of deliberate practice during the pubertal years). Often analysis of covariance is used to interpret the youth sports data, likely overfitting the data, as multiple comparisons are highly problematic using a single-level model, even more in settings with a low group-level variation (Gelman, Hill, & Yajima, 2012);

(ii) we examined the need to consider multilevel models by looking into null models (the simplest two level model which includes only the group-level parameters (also referred to as random parameters), to measure the proportion of total variance which fell between-groups (i.e., variance partition coefficient also reffered to as intracalss correlation)(Goldstein, 2011). Variance partition correlations higher than 0.05 indicate a substantial variation by the respective group and the need to use multilevel modeling (Goldstein, 2011; Snijders and Bosker, 2012).

(iii) the key feature of multilevel modelling is shrinkage (partial pooling), by incorporating group-level effects. It allows for more efficient use of the data. These may be understood as a weighted average between the all sample estimate and a group or unit estimate. The specific weights are based on the entire variation of the sample and the group variation (Gelman and Hill, 2007). In particular, the partial pooling will be stronger for smaller units with fewer observations (Buttice and Highton, 2013)

We agree with the argument to interpret the age effect assuming chronological age in the model. Indeed categorization removes potential variation. We choose to use age groups to echo the ecological context where players are grouped in competitive age groups.Also, following our previous point, partial pooling helps to mitigate the limitations of our choice for group age.

l207:
What do “population-level parameter” and “group-level parameters” mean? Is “population-level parameter” equivalent to beta? Is "group-level parameter" equivalent to alpha? If you called sigma "group-level parameter", it would be wrong. Sigma is often called “hyper parameter”. Alpha is often called “random intercept” or “varying intercept”.What did you set a prior for each sigma?
In addition, in the result section, you should report the results of inferred group-level intercept (i.e., beta) and hyper parameters (i.e., sigma).
Authors´ reply: We used referred to the hyperparameters as population-level effects, which are referred to as fixed effects in frequentist analysis, and group-level effects which are referred to as random effects in frequentist analysis. This is the common designation when using Bayesian analysis. We inserted the comparable frequentist terms in the text to allow the unfamiliar reader with Bayesian analysis to better understand our report. For the residual standard deviation parameter (level-1 standard deviation or referred to as residuals), we adopted the default prior in the brms package. For the group-level effects (i.e., level-2 standard deviations, in this case$\sigma_{a},\sigma_{m},\sigma_{d},\sigma_{g}$) we used half-normal priors, as we intend to have a conservative approach on the analysis by stating that the group-level estimates are unlikely to be greater than one standard deviation of the outcome.

Figures, Table, and Figure captions:
Figures and Table do not include enough information. You should also refine them.
Authors´reply: We reformatted the figures, dodging the estimates and uncertainty, as well as refined the legends. Hopefully, we appropriately address the reviewer´s comment.

In figure 1, what does each color of lines mean?
How did you produce the predicted lines in Figure 1? They seemed to be produced from a nonlinear regression model, but the details of the model were not found. Please explain what “the growth references of the WHO” indicates.

Authors´reply: We did not model the data from growth references of the World Health Organization or the US reference population (for body mass). We plotted the data as a comparison of the general population, which is available at both the WHO and CDC websites. We made available in the OSF repository all the data and codes that allow for replication of our procedures.

The colors indicate the percentiles of the reference growth data. We agree that this information should be explicit in the plot or captions. We added in the captions, to allow the plot to be cleaner.

In Figure 2-Figure 6, lines overlapped, and we could not detect each line. Each line should be displayed by shifting each position. In addition, I could not distinguish the 90% and 67% intervals in these Figures.
What does "mat_cat" mean?

Authors´reply: We thank the reviewer´s comment. Hopefully, we provide cleaner and clear Figures now. We dodged the estimates and uncertainty to remove the overlap. The legend titles were corrected in all figures, removing the variable label from the dataset.

l249-l253: I could not figure out which part we should see in each figure in order to understand “there was substantial variation between female and male players”.
Figure 6 is identical with Figure 5?

Authors´reply: We apologize for the incorrect upload of the figures, We have corrected appropriately both Figure 5 and Figure 6. The Bayesian inference allows for direct probabilistic comparisons between estimates and uncertainty. Hopefully, with the changes in the Figures, it becomes clear for the reader.

In Table 1, I could not understand what “1-5”, which was described in “Deliberate practice motivation” and “Achievement and competitiveness motivation”, meant.

Authors´reply: It represents the scale used in the questionnaires based on a Likert-like scale. The information about the questionnaires is provided in the methods section. It is a common practice to describe questionnaire scales, but we are open to suggestions to improve our reporting.

Reviewer #2: The authors provide a description of the variation in body size, functional capacities, motivation for achievement and competitiveness, and motivation for deliberate practice of 68F+94M Brazilian adolescent volleyball players. The paper essentially consists of a descriptive statistics (with a Bayesian multilevel modelling for estimates' adjustment), oriented toward the assessment of different levels of motivations. The manuscript is reasonably written, and quite easy to follow also by an audience of non-specialists; from a data science perspective the paper is quite simple and straightforward, with very basic statistical approaches.

Hereafter I list a few points that the authors need to take into account and discuss further:

- aim of the research needs to be better clarified - the descriptive purposes is rather vague and poorly focused

Authors´reply: We agree with the reviewer´s point. We re-phrased the purpose of the study.

- the use of the Bayesian modelling should be explained in more details, both in terms of motivation and in its mathematical application in the current situation

Authors´reply: We agree with the reviewer´s point. We added a section in the introduction presenting the need for multilevel modeling and Bayesian methods.

- the sample size is too small to allow for robust generalisation of the results - in the discussion the conclusions drawn are beyond the support provided by the shown results.

Authors´reply: We agree with the reviewer. At the end of the discussion, we argue that we discuss and speculate based on a single observational study, which warrants a conservative interpretation and generalization of the observations. Also, we believe Bayesian inference's main strength lies in the explicit updating of knowledge based on data accumulated from several observational studies, particularly in scientific areas using different sources and data levels such as sport sciences.

- the term "credible interval" is somehow non-standard; what's the mathematical definition of "credible"? I would suggest using the word "confidence" instead, specifying the statistics used (e.g. 95% bootstrap t-Student)

Authors´reply: In Bayesian analysis, credible interval reflects the interval of the posterior probability. These posterior intervals report two parameter values that contain between them a specified amount of posterior probability, a probability mass (McElreath, 2015), the range of parameter values compatible with the model and data. The term has been used interchangeably with confidence interval, and compatibility interval has been proposed lately as a solution (Amrhein, Greenland, & McShane, 2019). We followed the reviewer's suggestion and replaced the term credibility with confidence in the text, noting that both are used interchangeably in the context of Bayesian analysis.

- the usefulness of the results for volleyball trainers and specialists should be better pointed out, for instance providing some case studies highlighting how decision can be formulated by using the obtained outcomes.
Authors´reply: We thank the reviewer´s observation. We added interpretations applied to coaches and trainers. Importantly and about a previous comment, we allowed for some speculation with our data. Still, we acknowledged our interpretations' limits and the need to be conservative with a single observation study. We hope to have addressed the reviewer´s comments satisfactorily.

- the use of amateur level player can represent a limiting factor of the study; in the authors' opinion, what are the differences to be expected if pro-level athletes are used instead for the same study?

Authors´reply: We used two contexts of young volleyball players' development aiming for expertise. This context is comparable to sports academies in other sports in Europe, such as soccer or tennis, where young athletes devote their focus and resources to performance development. Hence, sport selection is likely key, and the factors influencing coaches decisions on athletes´ development (individual and contextual) may be represented in our sample. We agree with the reviewer that competitive level and training context likely influence the results. For example, at the school sports level, which in Brazil represent a relevant path of youth sports participation, players' characteristics (size, growth, maturation, performance, and behaviors) likely vary from the players in our sample and probably from the population we are interested in the present study.

References

Amrhein, V., Greenland, S., & McShane, B. (2019). Scientists rise up against statistical significance. *Nature, 567*(7748), pp. 305-307. doi:10.1038/d41586-019-00857-9 Retrieved from <http://www.ncbi.nlm.nih.gov/pubmed/30894741>

Buttice, M. K., & Highton, B. (2013). How Does Multilevel Regression and Poststratification Perform with Conventional National Surveys? *Political Analysis, 21*(4), pp. 449-467. doi:10.1093/pan/mpt017 Retrieved from https://[www.cambridge.org/core/article/how-does-multilevel-regression-and-poststratification-perform-with-conventional-national-surveys/113B73B46C65BFA037146C4A5FFDC7E9](http://www.cambridge.org/core/article/how-does-multilevel-regression-and-poststratification-perform-with-conventional-national-surveys/113B73B46C65BFA037146C4A5FFDC7E9)

Gelman, A., & Hill, J. (2007). *Data analysis using regression and multilevel/hierarchical models* Cambridge: Cambridge University Press.

Gelman, A., Hill, J., & Yajima, M. (2012). Why We (Usually) Don't Have to Worry About Multiple Comparisons. *Journal of Research on Educational Effectiveness, 5*(2), pp. 189-211. doi:10.1080/19345747.2011.618213 Retrieved from https://doi.org/10.1080/19345747.2011.618213

Goldstein, H. (2011). *Multilevel statistical models* (4th ed.) Chichester, West Sussex: Wiley.

McElreath, R. (2015). *Statistical rethinking : a Bayesian course with examples in R and Stan* Boca Raton, FL: Chapman & Hall/CRC Press.

Snijders, T. A. B., & Bosker, R. J. (2012). *Multilevel analysis : an introduction to basic and advanced multilevel modeling* (2nd ed.) Los Angeles: Sage.
